# Machine Learning for Prediction of Survival Outcomes with Immune-Checkpoint Inhibitors in Urothelial Cancer

**DOI:** 10.3390/cancers13092001

**Published:** 2021-04-21

**Authors:** Ahmad Y. Abuhelwa, Ganessan Kichenadasse, Ross A. McKinnon, Andrew Rowland, Ashley M. Hopkins, Michael J. Sorich

**Affiliations:** 1 College of Medicine and Public Health, Flinders University, Adelaide 5000, Australia; ganessan.kichenadasse@flinders.edu.au (G.K.); ross.mckinnon@flinders.edu.au (R.A.M.); andrew.rowland@flinders.edu.au (A.R.); ashley.hopkins@flinders.edu.au (A.M.H.); michael.sorich@flinders.edu.au (M.J.S.); 2 Department of Medical Oncology, Flinders Centre for Innovation in Cancer/Flinders Medical Centre, Adelaide 5000, Australia; 3 Cancer Clinical Network, Commission for Excellence and Innovation in Health, Adelaide 5000, Australia

**Keywords:** machine learning, survival outcomes, immune checkpoint inhibitors, gradient boosting, random forest

## Abstract

**Simple Summary:**

Machine learning (ML) is a form of artificial intelligence that could be used to enhance the efficiency of developing accurate prediction models for survival outcomes with cancer medicines, which is critical in informing disease prognosis and care planning. We used data from two recent clinical trials to develop and validate ML‐based clinical prediction models of the overall and progression‐free survival rates in patients with urothelial cancer initiating the immune checkpoint inhibitor (ICI) atezolizumab. We demonstrated that ML can efficiently develop an accurate prediction model of survival, enable an accurate prognostic risk classification, and provide realistic expectations of treatment outcomes in patients undergoing urothelial cancer-initiating ICIs therapy.

**Abstract:**

Machine learning (ML) may enhance the efficiency of developing accurate prediction models for survival, which is critical in informing disease prognosis and care planning. This study aimed to develop an ML prediction model for survival outcomes in patients with urothelial cancer-initiating atezolizumab and to compare model performances when built using an expert-selected (curated) versus an all-in list (uncurated) of variables. Gradient-boosted machine (GBM), random forest, Cox-boosted, and penalised, generalised linear models (GLM) were evaluated for predicting overall survival (OS) and progression-free survival (PFS) outcomes. C-statistic (c) was utilised to evaluate model performance. The atezolizumab cohort in IMvigor210 was used for model training, and IMvigor211 was used for external model validation. The curated list consisted of 23 pretreatment factors, while the all-in list consisted of 75. Using the best-performing model, patients were stratified into risk tertiles. Kaplan–Meier analysis was used to estimate survival probabilities. On external validation, the curated list GBM model provided slightly higher OS discrimination (c = 0.71) than that of the random forest (c = 0.70), CoxBoost (c = 0.70), and GLM (c = 0.69) models. All models were equivalent in predicting PFS (c = 0.62). Expansion to the uncurated list was associated with worse OS discrimination (GBM c = 0.70; random forest c = 0.69; CoxBoost c = 0.69, and GLM c = 0.69). In the atezolizumab IMvigor211 cohort, the curated list GBM model discriminated 1-year OS probabilities for the low-, intermediate-, and high-risk groups at 66%, 40%, and 12%, respectively. The ML model discriminated urothelial-cancer patients with distinctly different survival risks, with the GBM applied to a curated list attaining the highest performance. Expansion to an all-in approach may harm model performance.

## 1. Introduction

Urothelial cancer is an aggressive malignancy associated with about 200,000 global deaths annually and a 5-year survival rate of about 5% in the metastatic setting [1,2]. Immune-checkpoint inhibitors (ICIs) targeting the programmed death-1 (PD-1) pathway, such as atezolizumab and pembrolizumab, are an important emerging treatment option for metastatic urothelial cancer. Nonetheless, there is substantial heterogeneity in response to ICIs, and identifying individuals upfront that are most likely to respond to this treatment is a clinical priority [3].

Clinical prediction models of survival outcomes integrating clinicopathological predictors using data from large cohorts of patients may enable improved decision making and identify patients with different therapeutic prognoses [4]. While some prediction models for urothelial cancer exist in the literature [5,6,7], these models were developed using traditional statistical approaches (e.g., Cox regression), assessed a small number of predictors, and have insufficient evidence for clinical use, as predictors can differ significantly between cancer types or subtypes and treatments [8,9,10].

Machine learning (ML), a form of artificial intelligence, is an emerging alternative that may efficiently develop accurate clinical prediction models that can deal with high-dimensional data, and identify complex relationships between variables and outcomes that may be unidentifiable with traditional statistical approaches [11,12,13]. There were several recent breakthroughs demonstrating how using ML to rapidly interrogate complex data delivers a more efficient use of healthcare resources, including the detection of COVID-19 infection by ML interrogation of CT and X-ray images [14,15]. With respect to cancer, there are several ML-based algorithms that can process time-to-event survival outcome data, so identifying a suitable best-performing learning algorithm is critical to developing accurate prediction models of survival. To our knowledge, no study assessed ML-based algorithms’ ability to predict survival outcomes with immunotherapy in urothelial cancer. Furthermore, it is unclear whether developing ML prediction models using an all-in list (uncurated) of variables augments the discrimination performance of prediction models when compared to that of models developed with an expert preselected list (curated) (i.e., the traditional approach of selecting variables based on clinical knowledge and prior evidence). Therefore, the aims of this study were to develop and externally validate ML prediction models of survival outcomes for patients with urothelial-cancer-initiating atezolizumab and to compare the performance of ML models built with an expert-selected list (curated) versus an all-in list (uncurated) of variables.

## 2. Methods

### 2.1. Study Cohort

Individual participant data (IPD) from IMvigor210 (ClinicalTrials.gov Identifier: NCT02108652, data cut-off 4 July 2016) were used for model development (training set). IPD from the randomised atezolizumab arm of IMvigor211 (ClinicalTrials.gov Identifier: NCT02302807, data cut-off 13 March 2017) were used for external model validation (testing set). IMvigor210 was a single-arm Phase II trial in patients with locally advanced or metastatic urothelial cancer receiving atezolizumab 1200 mg IV every 3 weeks [2,16]. IMvigor211 was a Phase III trial in platinum-treated locally advanced or metastatic urothelial-cancer patients randomised to atezolizumab (1200 mg IV every 3 weeks) or chemotherapy (docetaxel (75 mg/m^2^ IV every 3 weeks), paclitaxel (175 mg/m^2^ IV every 3 weeks), or vinflunine (320 mg/m^2^ IV every 3 weeks) [17]. Data were accessed according to the Hoffmann–La Roche policy and made available through Vivli, Inc. (www.vivli.org).

IMvigor210 and IMvigor211 were conducted in accordance with the Guidelines for Good Clinical Practice and the Declaration of Helsinki [18]. Each participant signed and dated a written informed consent form before study enrolment [2,16,17]. Secondary analysis of deidentified IPD was classified as minimal-risk research and was confirmed as exempt from review by the Southern Adelaide Local Health Network, Office for Research and Ethics.

### 2.2. Predictors and Outcomes

The primary predicted outcome was overall survival (OS), with progression-free survival (PFS) as a secondary outcome. Primary study definitions of PFS were used, i.e., the independent review facility-assessed disease as per Response Evaluation Criteria in Solid Tumours (RECIST version 1.1) in IMvigor210, and the investigator-assessed disease as per RECIST (version 1.1) in IMvigor211.

Two lists of pretreatment variables were used to build the ML models: an expert preselected list (curated) and an all-in list (uncurated). The curated list included clinicopathological pretreatment factors (i.e., before the initiation of treatment) that were selected on the basis of biological plausibility, prior prognostic evidence, expert oncologist medical opinion, and being in line with prediction model guidelines for a minimum of 10 events per predictor variable [19]. The uncurated list included all pretreatment individual patient factors, subject to (1) being available in both training and validation data (≤10% missing), (2) available data definitions or reports for checking standardisation accuracy in a reasonable timeframe, and (3) within the top 15 most frequent comorbidities or concomitant medicines (i.e., an intention of the all-in approach was to speed up the model-building process rather than become stuck on needing an expert cancer researcher for data formatting and checking). Variables with missing values were imputed using approximate multiple imputation from the distribution of each variable conditional on all other variables as implemented in the transcan function from Hmisc package in R [20].

The curated list included 23 variables: (1) demographic factors (age, sex, race, body-mass index, and smoking status), (2) laboratory factors (levels of haemoglobin, albumin, white blood cells, C-reactive protein, neutrophil/lymphocyte ratio (NLR), platelet count, alkaline phosphatase, lactate dehydrogenase, and programmed death ligand 1 (PD-L1) expression on immune cells (PD-L1 ICs): PD-L1 IC0 (PD-L1 expression on <1%), PD-L1 IC1 (PD-L1 expression on ≥1% but <5%), and PD-L1IC2/3 (PD-L1 expression on ≥5) [2,16]), (3) disease or treatment factors (Eastern Cooperative Oncology Group Performance Status (ECOG-PS), disease stage (metastatic/locally advanced), the number of prior treatments, time since last chemotherapy, time since initial diagnosis, presence of liver metastasis, count of tumour sites, primary tumour site, and the presence of a urinary-tract infection.

The uncurated list contained 75 variables, composed of the curated-list variables and: (1) laboratory factors (derived NLR, eosinophil, neutrophils, alanine transaminase, aspartate aminotransferase, bilirubin, blood urea nitrogen, serum creatinine, estimated glomerular filtration rate, haemoglobin-to-platelet ratio, lymphocyte-to-monocyte ratio, platelet-to-lymphocyte ratio, and the total protein, calcium, potassium, sodium, magnesium, chloride, phosphate) and (2) disease or treatment factors (the presence of liver, brain, bone, lung or visceral metastasis, time since metastasis diagnosis, previous cystectomy, tumour stage at initial diagnosis, histologic characteristics, comorbidities (the presence of diabetes, hypertension, anaemia, renal failure, dyslipidaemia, fatigue, urinary-tract signs and symptoms, musculoskeletal pain, gastrointestinal and abdominal pain, generalised pain, constipation), and concomitant medicine use (opioids, antibiotics, proton-pump inhibitors, nonsteroidal anti-inflammatory drugs, statins, beta-blockers, calcium channel blockers, steroids, anticoagulants, anticonvulsant, bone-modulating agents, paracetamol, laxatives, vitamins, and minerals).

### 2.3. Machine-Learning Model Development

The patient cohort initiating atezolizumab in IMvigor210 was used for training the ML models. Four ML-based algorithms were evaluated: (1) gradient-boosted machine (GBM [21]), (2) random forest (RandomForestSRC [22]), (3) Cox-boosted model (CoxBoost [23]), and (4) penalised generalised linear models (GLM [24]). Hyperparameter tuning for ML algorithms was performed on the curated and uncurated lists by using grid search with 5-fold cross-validation with 10 repeats. Models were implemented in the ML package, mlr [25].

Model performance was externally validated using the independent IMvigor211 atezolizumab-treated patients. Model performance was assessed using the concordance statistic (c-statistic) [26] and calibration plots of observed versus predicted survival probabilities. The best-performing ML model was used to stratify patients into risk tertiles (low-, intermediate-, and high-risk prognostic groups). Survival probabilities of the risk groups were assessed using the Kaplan–Meier method. Relative variable importance in the best-performing ML model was evaluated using established methodologies [27,28]. All analyses were conducted in R version 3.6.2.

## 3. Results

### 3.1. Study Cohort

The training cohort consisted of 429 patients (239 OS events) who had initiated atezolizumab within IMvigor210. The validation cohort consisted of 467 patients (324 OS events) randomised to atezolizumab within IMvigor211. Patient characteristics are presented in Table S1. The median (95% CI) follow-up in IMvigor210 and IMvigor211 was 11 (11–12) and 17 (17–18) months, respectively.

### 3.2. Machine-Learning Model Development

**Figure 1:**
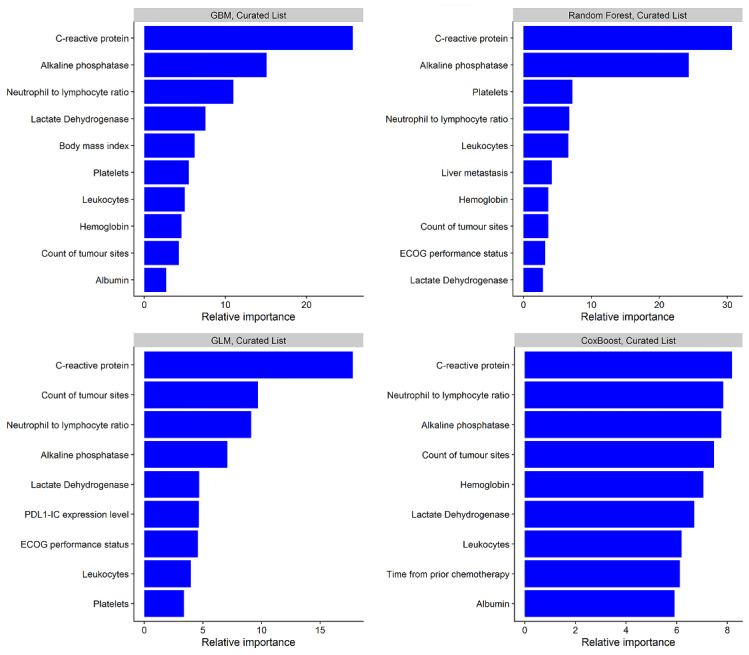
Relative importance of top 10 variables for predicting survival using machine-learning models built with the curated variable list. GLM: generalised linear model with regularisation. ECOG: Eastern Cooperative Oncology Group. PDL-1-IC: Programmed death-ligand 1 gene expression level on immune cells.

The search space and optimal hyperparameter values used for the developed ML models (both curated and uncurated versions) are presented in Table S2. The discrimination performance of the developed models on the training data is presented in Table S3 (both curated and uncurated versions). The top 10 most influential predictors of survival for each model (curated list versions) are presented in Figure 1, with C-reactive protein, alkaline phosphatase, neutrophil/lymphocyte ratio, lactate dehydrogenase, and the count of tumour sites among the most important variables in all constructed models. The top 10 most influential predictors from the developed models using the uncurated list are presented in Figure S1.

On external validation, the GBM algorithm provided slightly higher OS discrimination (c = 0.71) than that of random forest (c = 0.70), CoxBoost (c = 0.70), and GLM (c = 0.69) models (curated list versions) (Table 1). All models were equivalent in predicting PFS (c = 0.62). Expansion to the uncurated list was generally associated with slightly worse discrimination for the GBM (OS c = 0.70, PFS c = 0.62), random forest (OS c= 0.69, PFS c = 0.61), CoxBoost (OS c= 0.69, PFS c = 0.61), and GLM (OS c = 0.69, PFS c = 0.61) models (Table 1).

**Figure 2:**
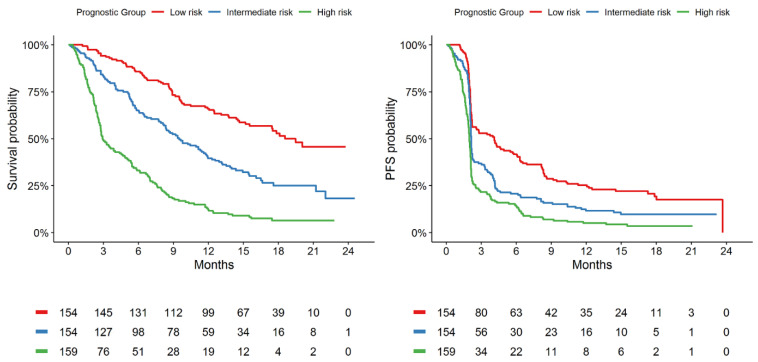
Kaplan–Meier estimates indicating differences in OS and PFS according to stratified machine-learning prognostic groups on validation cohort within IMvigor211 using curated-list GBM model.

On the basis of these observations, the GBM model built with the curated list was selected for further evaluation as it provided the highest and most consistent discrimination performance among the evaluated ML algorithms. On validation, the GBM model (curated-list version) was observed to be well-calibrated for OS and PFS prediction (Figure S2). The GBM model (curated-list version) was observed to discriminate 1-year OS probabilities of 66%, 40%, and 12% for the the low-, intermediate-, and high-risk groups, respectively, defined from the randomised atezolizumab patients of IMvigor211, while 1-year PFS probabilities of 25%, 12%, and 5% were observed for the defined risk groups (Table 2). Kaplan–Meir plots of OS and PFS probabilities are presented in Figure 2.

## 4. Discussion

In this study, we used data from two large, high-quality clinical trials and evaluated several ML algorithms in the development of prediction models of survival for patients with urothelial-cancer-initiating atezolizumab. The GBM algorithm consistently provided the highest OS validation performance using both the expert-selected (curated) and the all-in (uncurated) variable lists. However, expansion to the all-in list was associated with slightly worse discrimination performance compared to that in the expert-selected subset. The GBM model (curated list) was able to discriminate patients into three prognostic risk groups with distinct survival outcomes. To our knowledge, this is the first study to use ML‑based approaches to develop and validate a survival-prediction model in patients with urothelial-cancer-initiating immunotherapy, and the first study to compare the performance of ML models built with an expert-selected variable list versus an all-in approach.

ML was applied to cancer diagnosis and risk assessment, but minimally explored for predicting personalised survival outcomes with emerging ICIs [12,29,30,31]. Recently, Hopkins et al. assessed 24 predictor variables to develop an OS prediction model for patients with nonsmall-cell lung cancer (*n* = 797) treated with atezolizumab, and model-validation performance using the random-forest approach (c = 0.77) was found to be superior to the GLM (0.76) and ctree (c = 0.69) models [4]. Comparatively, our study evaluated a wider range of ML algorithms and externally validated them using a large independent cohort of patients.

In addition to comparing ML algorithms in a new cancer-treatment modality, this study demonstrates that ML is proficient at identifying important predictors of treatment outcomes with ICIs in urothelial cancer. In this analysis, ML identified C-reactive protein, alkaline phosphatase, neutrophil/lymphocyte ratio, lactate dehydrogenase, and the count of tumour sites among the most important variables in all constructed models, in agreement with previous research assessing atezolizumab therapeutic outcomes in nonsmall-cell lung cancer [4,32,33]. Further, the developed model may be able to facilitate accurate risk stratification based on individual patient characteristics. For example, on external validation in the atezolizumab arm of IMvigor211, the GBM model had prediction performance consistent with a strongly performing model (c = 0.71) [8,34], and it was able to discriminate patients into low-, intermediate-, and high-risk groups with estimated 1-year OS probabilities of 66%, 40%, and 12%, respectively. This demonstrates the potential of ML prediction models to inform treatment decisions and provide more realistic expectations for treatment outcomes with patients initiating ICIs.

Expansion to the all-in (uncurated) variable-list approach resulted in slightly worse prediction performance. The slight deterioration in performance may have been due to the presence of noninformative variables that ultimately cause model overfitting or uncertainty [35]. While the all-in (dump-and-play) approach has the potential to enable biostatisticians to begin model building without expert input, the time required for artificial intelligence to tune and fit the model was substantially longer than the time required to tune the model using the curated list with fewer variables. Ultimately, it was our experience that reducing the variable list with expert help both improved model performance and saved time from a computational perspective.

A strength of this analysis was the completeness and quality of the large contemporary immunotherapy dataset that was used to train and then externally validate model discrimination and calibration performance. In addition, we studied two outcomes, OS and PFS, and we were able to confirm the insights about ML performance for each outcome. Regarding the all-in list, it is possible that some variables were not collected in the IMvigor210 and IMvigor211 trials, and the nature of clinical-trial inclusion criteria can limit the generalisability of data distributions when compared to routine care. As the model developed and validated in this study used data from the IMvigor210 and IMvigor211 trials, the training and validation cohorts were restricted to patients with urothelial-cancer-initiating atezolizumab monotherapy. Confirming the performance of ML prediction models for other ICIs, ICI combination therapy, anticancer-medicine classes, lines of therapy, and cancer types is an important future direction.

## 5. Conclusions

Using two large, contemporary clinical trials, we demonstrated that the GBM algorithm, applied to an expert-selected variable list, attained the highest validation performance for OS prediction. This model was further demonstrated capable of discriminating urothelial-cancer patients initiating atezolizumab with distinctly different survival outcomes. Using an all-in list of variables as opposed to an expertly selected list may harm discrimination performance.

## Figures and Tables

**Table 1:** Discrimination performance on validation set for overall survival (OS) and progression-free survival (PFS).

	OS		PFS	
	Curated list	Uncurated list	Curated list	Uncurated list
Learner	C-statistics	C-statistics	C-statistics	C-statistics
Gradient-boosted machine	0.71	0.70	0.62	0.62
Random forest	0.70	0.69	0.62	0.61
Cox-boosted	0.70	0.69	0.62	0.61
generalised linear model	0.69	0.69	0.62	0.61

**Table 2:** Effect size and 1-year survival probability for gradient-boosted machine (GBM) stratified prognostic groups on validation cohort within IMvigor211.

Prognostic Group	OS		PFS	
	HR (95% CI) *(p < 0.001)*	1-year survival probability (95% CI)	HR (95% CI) *(p < 0.001)*	1-year PFS probability (95% CI)
Low risk	1.00	66% (59–74)	1.00	25% (19–33)
Intermediate risk	2.09 (1.55–2.81)	40% (33–48)	1.54 (1.21–1.98)	12% (7–18)
High risk	5.09 (3.81–6.81)	12% (8–19)	2.38 (1.87–3.04)	5% (3–10)

## Data Availability

Data were accessed according to Roche’s policy and process for clinical study data sharing and is available for request at https://vivli.org/.

## Supplementary Materials

The following are available online at https://www.mdpi.com/article/10.3390/cancers13092001/s1, Figure S1: Relative importance of the top 10 variables for predicting survival using the uncurated variable list. GBM: gradient boosted machine, GLM: generalized linear model with regularization, Figure S2: Calibration plots (Kaplan-Meier observed versus model predicted probabilities) of overall survival (OS) and progression free survival (PFS) on validation data using the GBM model constructed using curated variables list, Table S1: Pre-treatment patient characteristics by atezolizumab study cohort, Table S2: Hyperparameter tuning of machine learning algorithms on overall survival data using the primary and extended lists, Table S3: Discrimination performance on IMvigor210 training cohort for overall survival and progression free survival.
