# Supplementary material for: Machine Learning for Prediction of Survival Outcomes with Immune-Checkpoint Inhibitors in Urothelial Cancer
Source: Cancers (Basel). 2021 Apr 21;13(9):2001. doi: 10.3390/cancers13092001 (PMC8122430; doi:10.3390/cancers13092001)
Supplement: Supplementary file 1 [file cancers-13-02001-s001.zip › cancers-1176915-supplementary.pdf]

cancers

MDPI

Article

Supplementary Materials: Machine Learning for Prediction of Survival Outcomes with Immune Check Point Inhibitors in Urothelial Cancer

Ahmad Y. Abuhelwa, Ross A. McKinnon, Andrew Rowland, Ashley M. Hopkins and Michael J. Sorich

Table S1: Pre-treatment patient characteristics by atezolizumab study cohort.

Variable	Total No. = 896	IMvigor210 No. = 429	IMvigor210 No. = 467	*p*-value
PD-L1 tumor cell expression level				0.009
0	688 (77%)	347 (81%)	341 (73%)	
1	80 (9%)	30 (7%)	50 (11%)	
2	102 (11%)	38 (9%)	64 (14%)	
3	26 (3%)	14 (3%)	12 (3%)	
Aspartate Aminotransferase (U/L)				0.80
Median (IQR)	21 (16–26)	21 (16–27)	20 (16–26)	
Missing	33 (4%)	12 (3%)	21 (4%)	
Alanine Aminotransferase (U/L)				0.002
Median (IQR)	18 (13–26)	19 (14–28)	17 (12–25)	
Missing	35 (4%)	15 (3%)	20 (4%)	
Bilirubin (μmol/L)				0.054
Median (IQR)	6.8 (5.1–9.0)	6.8 (5.1–10.3)	6.8 (5.1–8.6)	
Missing	34 (3.8%)	13 (3.0%)	21 (4.5%)	
Neutrophils (10^9/L)				0.63
Median (IQR)	4.9 (3.7–6.5)	4.9 (3.8–6.5)	4.9 (3.6–6.6)	
Missing	29 (3.2%)	11 (2.6%)	18 (3.9%)	
Eosinophils (10^9/L)				0.011
Median (IQR)	0.15 (0.10–0.23)	0.17 (0.10–0.25)	0.13 (0.10–0.21)	
Missing	92 (10.27%)	46 (10.72%)	46 (9.85%)	
Lymphocyte to monocyte ratio (10^9/L)				0.002
Median (IQR)	2.1 (1.4–3.0)	2.0 (1.3–2.8)	2.3 (1.5–3.0)	
Missing	33 (3.7%)	12 (2.8%)	21 (4.5%)	
Derived neutrophil to lymphocyte ratio (10^9/L)				0.038
Median (IQR)	2.3 (1.7–3.1)	2.3 (1.8–3.2)	2.2 (1.7–3.0)	
Missing	34 (3.8%)	14 (3.3%)	20 (4.3%)	
Platelet to lymphocyte ratio (10^9/L)				0.006
Median (IQR)	193 (137–279)	204 (142–291)	182 (131–262)	
Missing	37 (4%)	15 (3%)	22 (5%)	
Hemoglobin to platelet ratio (g/L)				0.65
Median (IQR)	0.49 (0.36–0.64)	0.49 (0.36–0.66)	0.51 (0.37–0.63)	
Missing	27 (3.01%)	11 (2.56%)	16 (3.43%)	
Protein (g/L)				0.004
Median (IQR)	72 (68–76)	71 (67–75)	73 (69–76)	
Missing	38 (4%)	15 (3%)	23 (5%)	

Brain tumor site at baseline	8 (1%)	8 (2%)	0 (0%)	0.009
Bone tumor site at baseline	185 (21%)	75 (17%)	110 (24%)	0.031
Lung tumor site at baseline	359 (40%)	174 (41%)	185 (40%)	0.83
Visceral tumor site	682 (76%)	321 (75%)	361 (77%)	0.43
Estimated glomerular filtration rate (mL/min)				0.047
Median (IQR)	64 (50–81)	62 (47–80)	66 (51–82)	
Missing	43 (5%)	16 (4%)	27 (6%)	
Creatinine (μmol/L)				<0.001
Median (IQR)	103 (87–126)	107 (88–133)	100 (84–121)	
Missing	30 (3%)	12 (3%)	18 (4%)	
Blood Urea Nitrogen (mmol/L)				0.020
Median (IQR)	7.1 (5.7–9.0)	7.5 (5.8–9.3)	6.8 (5.7–8.6)	
Missing	51 (5.7%)	15 (3.5%)	36 (7.7%)	
Months from metastatic diagnosis (months)				0.20
Median (IQR)	9.2 (3.8–16.7)	8.6 (3.3–17.4)	9.9 (5.0–16.5)	
Missing	80 (8.9%)	30 (7.0%)	50 (10.7%)	
Histology				0.68
Transitional cell carcinoma	811 (91%)	386 (90%)	425 (91%)	
Transitional cell carcinoma with mixed histology	85 (9%)	43 (10%)	42 (9%)	
Prior cystectomy	355 (40%)	156 (36%)	199 (43%)	0.065
Sodium (mmol/L)				0.31
Median (IQR)	139 (136–141)	138 (136–141)	139 (137–141)	
Missing	28 (3%)	11 (3%)	17 (4%)	
Calcium (mmol/L)				0.24
Median (IQR)	2.4 (2.3–2.4)	2.4 (2.2–2.4)	2.4 (2.3–2.4)	
Missing	30 (3.3%)	11 (2.6%)	19 (4.1%)	
Potassium (mmol/L)				0.074
Median (IQR)	4.4 (4.1–4.7)	4.3 (4.1–4.6)	4.4 (4.1–4.7)	
Missing	28 (3.1%)	11 (2.6%)	17 (3.6%)	
Magnesium (mmol/L)				0.47
Median (IQR)	0.82 (0.74–0.88)	0.82 (0.74–0.89)	0.82 (0.74–0.87)	
Missing	58 (6.47%)	27 (6.29%)	31 (6.64%)	
Chloride (mmol/L)				0.58
Median (IQR)	102 (99–104)	102 (99–105)	102 (99–104)	
Missing	41 (5%)	14 (3%)	27 (6%)	
Phosphate (mmol/L)				<0.001
Median (IQR)	1.1 (0.9–1.2)	1.1 (0.9–1.2)	1.0 (0.9–1.2)	
Missing	54 (6.0%)	30 (7.0%)	24 (5.1%)	
Steroids (Y/N)	67 (7%)	40 (9%)	27 (6%)	0.059
Opioid (Y/N)	344 (38%)	176 (41%)	168 (36%)	0.14
NSAIDS (Y/N)	272 (30%)	141 (33%)	131 (28%)	0.14
Antibiotics 60 day PRE (Y/N)	70 (8%)	34 (8%)	36 (8%)	1.0
Proton pump inhibitors (Y/N)	241 (27%)	122 (28%)	119 (25%)	0.36
Paracetamol (Y/N)	287 (32%)	138 (32%)	149 (32%)	0.99
Laxatives or stool sonteners (Y/N)	220 (25%)	128 (30%)	92 (20%)	<0.001
Statins (Y/N)	215 (24%)	129 (30%)	86 (18%)	<0.001
Betablockers (Y/N)	171 (19%)	101 (24%)	70 (15%)	0.002
Vitamines and and minerals	190 (21%)	149 (35%)	41 (9%)	<0.001
Calcium channel blockers (Y/N)	141 (16%)	79 (18%)	62 (13%)	0.044

Anticoagulants (Y/N)	131 (15%)	70 (16%)	61 (13%)	0.20
Bone modulating agents (Y/N)	6 (1%)	4 (1%)	2 (<1%)	0.61
Insomnia (Y/N)	122 (14%)	70 (16%)	52 (11%)	0.031
Diabetes (all types) (Y/N)	138 (15%)	75 (17%)	63 (13%)	0.12
Anemia (Y/N)	159 (18%)	93 (22%)	66 (14%)	0.004
Pain and discomfort (Y/N)	453 (51%)	231 (54%)	222 (48%)	0.069
Vascular hypertension (Y/N)	452 (50%)	249 (58%)	203 (43%)	<0.001
Lipids metabolism disorders (elevated TG, Cholesterol, Hyperlipidemias) (Y/N)	249 (28%)	153 (36%)	96 (21%)	<0.001
Fatigue (Y/N)	228 (25%)	150 (35%)	78 (17%)	< 0.001
Constipation (Y/N)	212 (24%)	123 (29%)	89 (19%)	<0.001
Renal failure and impairment (Y/N)	103 (11%)	70 (16%)	33 (7%)	<0.001
Urinary tract signs and symptom (Y/N)	106 (12%)	67 (16%)	39 (8%)	0.001
Muscuskeletal pain (Y/N)	216 (24%)	126 (29%)	90 (19%)	<0.001
Gastrointestinal and abdominal pain (excluding oral/throat) (Y/N)	79 (9%)	46 (11%)	33 (7%)	0.070

Data are median (IQR) or number of patients (%). *p* values per Chi-Square test for categorical data and Kruskal-Wallis test for continuous data.

Table S2. Hyperparameter tuning of machine learning algorithms on overall survival data using the primary and extended lists.

ML Method	Parameter	Description	Search Space	Optimal Model / Parameter Value	
				Curated List	Uncurated List
Decision tree	teststat	The type of the test statistic to be applied for variable selection.	quadratic, maximum	'quadratic'	'quadratic'
	testtype	Method to compute the distribution of the test statistic.	Bonferroni, MonteCarlo, Univariate, Teststatistic	'MonteCarlo'	'Bonferroni'
	maxdepth	Maximum depth of the tree.	5,10,15,20	15	5
Random forest	ntree	number of trees in the forest	1000,2000,3000	2000	1000
	mtry	Number of variables randomly selected for splitting at each node.	4,5,6,8,10	4	4
	nodesize	Minimum size of terminal nodes.	5,10,15,20	5	15
	nsplit	Number of randomly selected split points.	5,10,15,20	5	5
Gradient boosted machine	Shrinkage	Learning rate	0.001–0.1, by = 0.001	0.001	0.001
	n.trees	Number of boosted trees	3000,4000,5000,6000	3000	4000
	Interaction.depth	Maximum depth of each tree	3,6,10	3	3
	n.minobsinnode	Minimum number of observations in the terminal nodes of the trees.	2–14, by = 2	8	10
	bag.fraction	Fraction of training set randomly selected to propose the next tree in expansion	0.5,0.8,1	0.5	0.5
Cox boosted model	Stepno	Number of boosting steps	50–200, by = 5	115	70
Generalized linear models	alpha	The elastic net mixing parameter	0,0.5,1	0	0

Table S3. Discrimination performance on IMvigor210 training cohort for overall survival and progression free survival.

Learner	Overall Survival		Progression Free Survival	
	Curated List C-Statistics	Uncurated List C-Statistics	Curated List C-Statistics	Uncurated List C-Statistics
Gradient boosted machine	0.81	0.84	0.70	0.72
Random Forest	0.88	0.83	0.73	0.71
Cox Boosted model	0.77	0.77	0.67	0.67
Generalized linear model	0.77	0.77	0.68	0.68

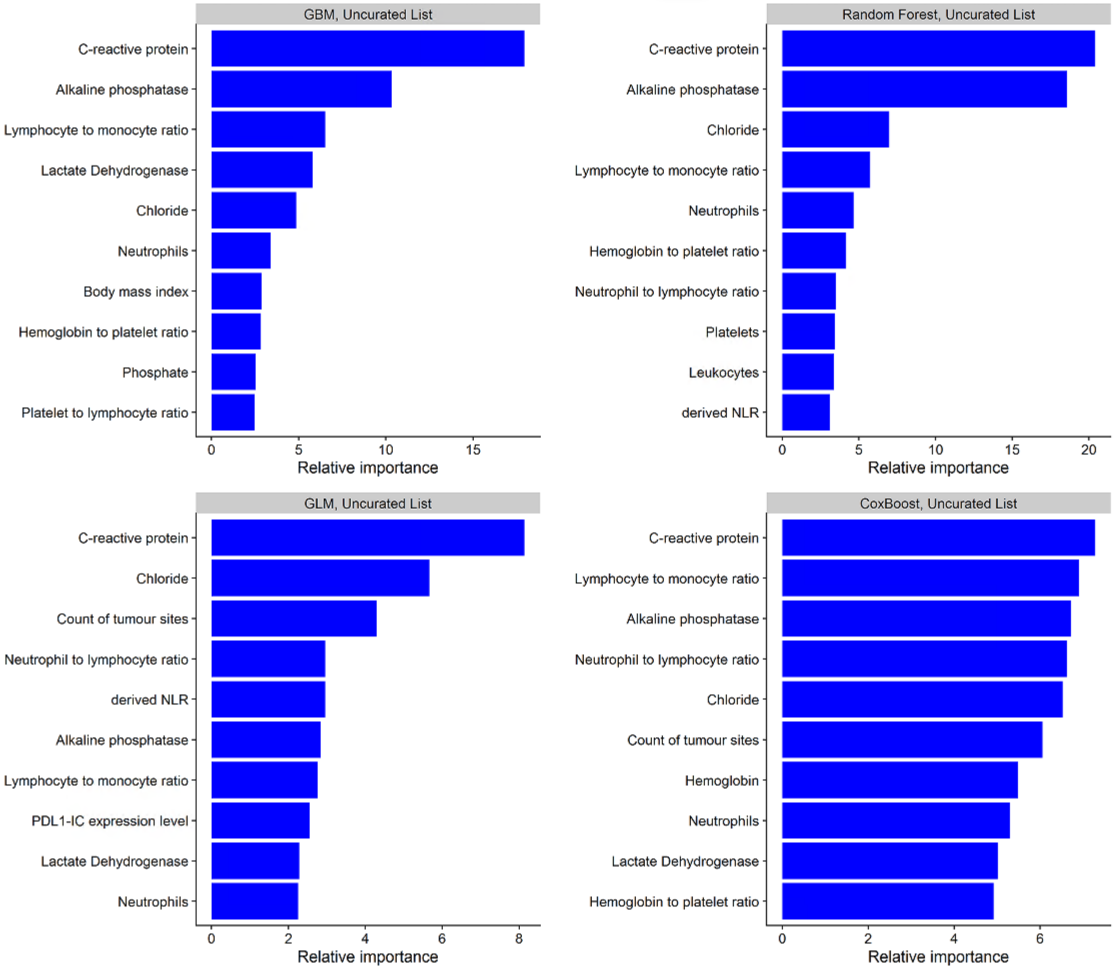

Figure S1. Relative importance of the top 10 variables for predicting survival using the uncurated variable list. GBM: gradient boosted machine, GLM: generalized linear model with regularization.

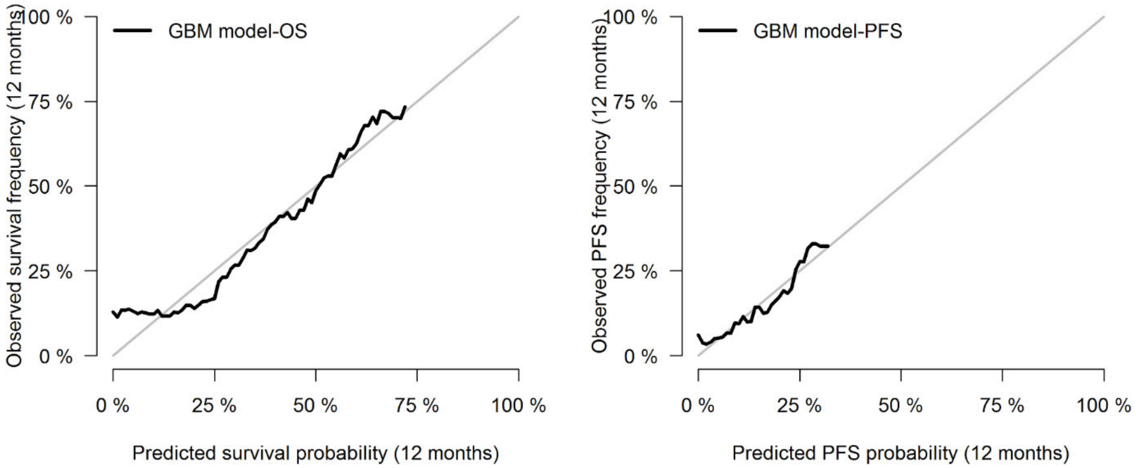

Figure S2. Calibration plots (Kaplan-Meier observed versus model predicted probabilities) of overall survival (OS) and progression free survival (PFS) on validation data using the GBM model constructed using curated variables list.
